# Camphene Attenuates Skeletal Muscle Atrophy by Regulating Oxidative Stress and Lipid Metabolism in Rats

**DOI:** 10.3390/nu12123731

**Published:** 2020-12-03

**Authors:** Suji Baek, Jisu Kim, Byung Seok Moon, Sun Mi Park, Da Eun Jung, Seo Young Kang, Sang Ju Lee, Seung Jun Oh, Seung Hae Kwon, Myung Hee Nam, Hye Ok Kim, Hai Jeon Yoon, Bom Sahn Kim, Kang Pa Lee

**Affiliations:** 1 Research and Development Center, UMUST R&D Corporation, Seoul 05029, Korea; u-service@naver.com; 2 Physical Activity and Performance Institute, Konkuk University, Seoul 05029, Korea; kimpro@konkuk.ac.kr; 3 Department of Nuclear Medicine, Ewha Womans University College of Medicine, Seoul 07804, Korea; psm9728@ewhain.net (S.M.P.); kkellydaeun92@naver.com (D.E.J.); eironn02@gmail.com (S.Y.K.); hyeok1003@hanmail.net (H.O.K.); haijeon.yoon@gmail.com (H.J.Y.); 4 Department of Nuclear Medicine, Asan Medical Center, University of Ulsan College of Medicine, Seoul 05505, Korea; atlas425@amc.seoul.kr (S.J.L.); sjoh@amc.seoul.kr (S.J.O.); 5 Seoul Center, Korean Basic Science Institute, Seoul 02841, Korea; kwonsh@kbsi.re.kr (S.H.K.); nammh@kbsi.re.kr (M.H.N.)

**Keywords:** oxidative stress, muscle atrophy, lipid metabolism, camphene, sarcopenia

## Abstract

Sarcopenia- or cachexia-related muscle atrophy is due to imbalanced energy metabolism and oxidative stress-induced muscle dysfunction. Monoterpenes play biological and pharmacological reactive oxygen species (ROS) scavenging roles. Hence, we explored the effects of camphene, a bicyclic monoterpene, on skeletal muscle atrophy in vitro and in vivo. We treated L6 myoblast cells with camphene and then examined the ROS-related oxidative stress using Mito Tracker^TM^ Red FM and anti-8-oxoguanine antibody staining. To investigate lipid metabolism, we performed real-time polymerase chain reactions, holotomographic microscopy, and respiratory gas analysis. Rat muscle atrophy in in vivo models was observed using ^18^F-fluoro-2-deoxy-D-glucose positron emission tomography/computed tomography and immunocytochemistry. Camphene reversed the aberrant cell size and muscle morphology of L6 myoblasts under starvation and in in vivo models. Camphene also attenuated E3 ubiquitin ligase muscle RING-finger protein-1, mitochondrial fission, and 8-oxoguanine nuclear expression in starved myotubes and hydrogen peroxide (H_2_O_2_)-treated cells. Moreover, camphene significantly regulated lipid metabolism in H_2_O_2_-treated cells and in vivo models. These findings suggest that camphene may potentially affect skeletal muscle atrophy by regulating oxidative stress and lipid metabolism.

## 1. Introduction

Muscle atrophy is characterized by the loss of muscle mass and concurrent muscle dysfunction, leading to limited muscular endurance, cardiorespiratory function, flexibility, balance, and quickness [1]. While sarcopenia is a type of muscle atrophy that naturally occurs with aging, cachexia is a muscle disorder induced by diverse pathogenic conditions, including immunodeficiency diseases and cancer [2]. To prevent muscle atrophy, the ingestion of appropriate doses of essential amino acid-centered protein combined with strength training and aerobic exercise is effective [3]. Muscle deficiencies, such as atrophy caused by sarcopenia or cachexia, are treated by identifying and eliminating the etiological factors causing the symptoms and the concomitant disease. However, effective pharmacological therapeutic agents that can reverse muscle atrophy have not yet been developed.

Muscle atrophy caused by sarcopenia or cachexia affects the energy balance and the extent of muscle protein degradation, consequently increasing mortality by exacerbating chronic disease [4]. Especially, the regulation of reactive oxygen species (ROS) generation and stabilization of energy metabolism may be promising strategies for controlling skeletal muscle atrophy [5]. In skeletal muscle, mitochondrial dysfunction, and ROS generation are key mechanisms in atrophy progression [6]. Although the physiological level of ROS in a cell is involved in signal pathways, such as differentiation, growth, and contraction, excessive ROS content due to cellular or environmental stress is the key triggering the pathological mechanisms and disease [7,8]. Some reports demonstrated that ROS-mediated induction of muscle RING-finger-1 (MuRF-1) and atrogin-1 are required to activate muscle protein breakdown and reduce muscle mass [9,10]. In addition, pathological accumulation of lipid accelerates muscle atrophy and myosteatosis [11]. Glucose production is activated by enzymes, such as glucose transporter type 4 (Glut4) and monocarboxylate transporter 1 (MCT1), to maintain energy sources in all mammalian cells [12,13]. The fatty acid translocase/CD36, carnitine palmitoyltransferase 1 (CPT1), and acetyl-CoA carboxylase 1 (ACC1) are important enzymes for fatty acid oxidation in the muscles [14,15]. Therefore, it is important to distinguish the glucose/fat mechanisms underlying atrophy to gain clinical control of the disease.

Diverse studies have reported that essential oils have beneficial pharmaceutical effects against oxidative stress, melanosis, and skin damage [16]. Camphene, a monoterpene found in essential oils EOs, is a candidate for the treatment of skeletal muscle atrophy as it regulates lipid accumulation in HepG2 cells and hyperlipidemia rats [17]. In the present study, we hypothesized that camphene could reduce oxidative stress during skeletal muscle atrophy progression by regulating ROS generation. We evaluated cell viability, ROS generation, and lipid metabolism after camphene treatment to investigate this hypothesis. We also investigated methods for evaluating muscle atrophy in vivo using the molecular imaging techniques, positron emission tomography/computed tomography (PET/CT), after intravenous injection of ^18^F-fluoro-2-deoxy-D-glucose (^18^F-FDG). Our research aims to investigate whether camphene can modulate ROS-induced skeletal muscle atrophy.

## 2. Materials and Methods

### 2.1. Materials

Materials for cell culture were purchased from Thermo Fisher Scientific (Waltham, MA, USA). The 2,3-bis-(2-methoxy-4-nitro-5-sulfophenyl)-2*H*-tetrazolium-5-carboxanilide (XTT) kit was purchased from Welgene Inc. (Gyeongsangbuk-do, Korea). Rat skeletal muscle cells (L6 cells) were obtained from the Korean Cell Line Bank (Seoul, Korea). Mito Tracker^TM^ Red FM was purchased from Thermo Fisher (Waltham, MA, USA). Anti-8-oxoguanine antibody was purchased from Merck (Burlington, MA, USA). All other reagents were purchased from Sigma-Aldrich (St. Louis, MO, USA).

### 2.2. Cell Viability and Cell Morphology Observation 

Cells were cultured, as previously described [18]. L6 cells were grown in high-glucose Dulbecco’s modified Eagle’s medium (DMEM) containing 10% fetal bovine serum (FBS) and 1% penicillin-streptomycin at 37 ± 2 °C and 5% CO_2_. Cells (1 × 10^4^ cells/well) were seeded in 96-well plates and treated with camphene (0–1000 μM) for 24 h. Cells were then incubated with XTT reagent for 2 h at 37 ± 2 °C, and then cell viability was measured on an iMARK microplate reader (Bio-Rad, Hercules, CA, USA) at 450 nm. Cell morphology was observed using light microscopy and holotomographic microscopy. Cell size was analyzed using ImageJ version 1.52a (United States National Institutes of Health, Bethesda, MD, USA).

### 2.3. Immunocytochemistry

L6 skeletal muscle cells (5 × 10^3^ cells/mL) were seeded in 8-well chambers for 24 h, then incubated in the absence or presence of serum and treated with camphene (300 μM) for 48 h. The cells were fixed and permeabilized in 4% formalin and 0.1% Triton X-100 for 10 min, then sequentially incubated with anti-MuRF-1 antibody (1:1000) and Alexa Fluor 488-conjugated secondary antibody (excitation: 492 nm, emission: 527 nm) for 1 h. To detect cellular ROS-involved oxidative stress, cells were incubated with Mito Tracker^TM^ Red FM (excitation: 581 nm, emission: 644 nm) and anti-8-oxoguanine (8-OXG) antibody for 30 min at 37 ± 2 °C. Images were obtained by fluorescence microscopy (Laser scanning microscopes 780, Ziess, Oberkochen, Germany). The nucleus was stained with 4′,6-diamidino-2-phenylindole (excitation: 358 nm, emission: 461 nm). Fluorescence intensity was measured and analyzed using ImageJ. Briefly, fluorescence was converted to gray after color separation and region of interest (ROI) values were defined as measured values of all gray levels.

### 2.4. Real-Time Polymerase Chain Reaction (PCR)

Total RNA was isolated from L6 skeletal muscle cells using TRIzol reagent, according to the manufacturer’s instructions. The Superscript III First Strand cDNA synthesis kit (Invitrogen, Carlsbad, CA, USA) was used to generate cDNA from 1 μg total RNA. Real-time PCR was performed on an Applied Biosystems 7500 Fast Real-Time PCR System (Thermo Fisher Scientific, Waltham, MA, USA) with SYBR Green PCR mix. Amplification involved an initial denaturation at 95 °C for 10 min followed by 40 cycles of denaturation at 95 °C for 10 s, annealing at 60 °C for 30 s, and extension at 72 °C for 30 s. The primers used are listed in Table 1. Relative mRNA levels were calculated using the 2^−ΔΔCt^ method and normalized to β-actin.

### 2.5. Animal Care and Starvation Protocols for PET/CT Imaging

The study was conducted in accordance with the Declaration of Helsinki, and the protocol was approved by the Ethics Committee of the Ewha Womans University College of Medicine Institutional Animal Care and Use Committee (EUM20-025, 22 April 2020). Male 8-week-old Sprague-Dawley rats were purchased from Orient Bio Inc. (Gyeonggi-do, Korea). The rats were individually housed with ad libitum access to water and food (AIN 93G formula) in a controlled environment (room temperature (24 ± 2 °C); humidity 40 ± 2%; 12 h light/dark cycle). Rats were divided into three groups (*n* = 4 each), which were either untreated group (UN), the group of the starved for 48 h and orally administered 1% Tween-80/saline once daily (ST); or the group of the starved for 48 h and orally administered camphene (6.8 mg/kg) in 1% Tween-80/saline once daily (ST + CA). The ST and ST + CA were provided free access to water during the starvation period. All rats in each group were imaged by ^18^F-FDG-PET, and their gastrocnemius muscles were dissected and isolated for histological analysis.

### 2.6. Small Animal PET-CT Imaging Protocol and Image Analysis

Animals received a single dose of ^18^F-FDG (22.9 ± 2.5 MBq) via intravenous tail injection. After 60 min in a temperature-controlled cage, rats were anesthetized with 2.5% isoflurane in a 7:3 mixture of N_2_/O_2_, and sequential PET-CT scans were acquired for 20 min in a dedicated small animal PET/CT scanner (NanoPET/CT, Mediso Medical Imaging Systems, Budapest, Hungary) with an 8.0-cm axial field-of-view (FOV) and a 10.0-cm transaxial FOV. CT scans were used for attenuation correction and anatomical localization of PET signals. Acquired images were reconstructed using the 3D Adjoint Monte Carlo method, which included scatter and random corrections. A 1.5-mm radius region of interest, including both mid-lower legs, was delineated by the intensely visualized region in the summed image. Regional uptake of radioactivity on both sides was decay-corrected to the injection time and expressed as the average standardized uptake value (SUV), which was normalized to the amount of radioactivity injected and the animal’s body weight. PET image analysis was performed using InterView Fusion software v3.03.089.0000 (Mediso Medical Imaging Systems, Budapest, Hungary). For the measurement of skeletal muscle masses on CT, the parameters were measured according to previously described literature [19]. The index of muscle mass (IMM) was defined as the ratio (T/L) between the thickness of the muscle (T) and the length of the tibia (L) acquired by MIM software v6.8.7 (MIM Software Inc., Cleveland, OH, USA). For each rat, the left and right legs were analyzed and averaged.

### 2.7. Histochemistry and Immunochemistry

Histochemistry and immunochemistry were performed as previously described [18]. The gastrocnemius muscles were fixed in 4% formalin, then sections were embedded in paraffin and serially sectioned into 5-μm slices. The prepared sections were cleared with xylene and hydrated with an ethanol gradient (70, 80, and 90%). To examine the histological change of muscle size, we performed hematoxylin and eosin staining. The stained specimen was analyzed with a microscope at 200× magnification, and five representative images (one central and four peripheral) were obtained from muscle sections, which were recorded using a CCD camera. Relative muscle size was analyzed by counting the number of muscles in the magnification fields. Some sections were incubated with anti-MuRF-1 primary antibodies overnight at 4 °C and then with Alexa Fluor 488-conjugated secondary antibodies for 1 h. Images were obtained by inverted and fluorescence microscopy, at excitation and emission wavelengths of 492 nm and 527 nm, respectively. Fluorescence intensity was measured using ImageJ.

### 2.8. Animal Care and Starvation Protocols for Energy Metabolism Experiments

Rats (*n* = 24) were adapted for 1 week in the same plastic cages. Eight rats were randomly selected to compare with the starvation group prior to this experiment, to measure energy consumption during the day. During this period, water and feed were supplied ad libitum. The remainder of the rats were divided into the ST (*n* = 8, starved and administered 1% Tween-80/saline), and the ST + CA (*n* = 8, starved, and orally administered 6.8 mg/kg camphene in 1% Tween-80/saline). Starvation was performed for 3 d, with only water supplied. Camphene and control treatments were administered daily at 08:00 during the starvation period.

### 2.9. Energy Metabolism Measurements

The respiratory gas analysis was conducted using an open circuit respiratory system, as in previous studies [20,21,22]. Rat O_2_ uptake and CO_2_ production were measured using a mass analyzer (model RL-600, Alco Systems, Chiba, Japan) and a switching system (model AN16-A-S, Alco Systems, Chiba, Japan), using eight acrylic metabolic chambers (23 × 10 × 13 cm^3^; chamber volume of approximately 3 L). The flow rate within the chamber was 3 L/min. Air from each chamber passed through a 6-mm diameter acrylic tube 3 m in length and was sampled for 15 s intervals.

### 2.10. Statistical Analysis

Statistical analysis was performed as previously described [23], in GraphPad Prism ver. 4.00 for Windows (GraphPad Software, La Jolla, CA, USA). The results are expressed as the mean ± standard deviation of at least three independent experiments (*n* ≥ 3). Between-group differences were determined using Student’s *t*-test and one-way analysis of variance. Tukey’s test was used for multiple comparisons. *p <* 0.05 was considered statistically significant.

## 3. Results

### 3.1. The Effect of Camphene on the Viability of L6 Skeletal Muscle Cells

**Figure 1:**
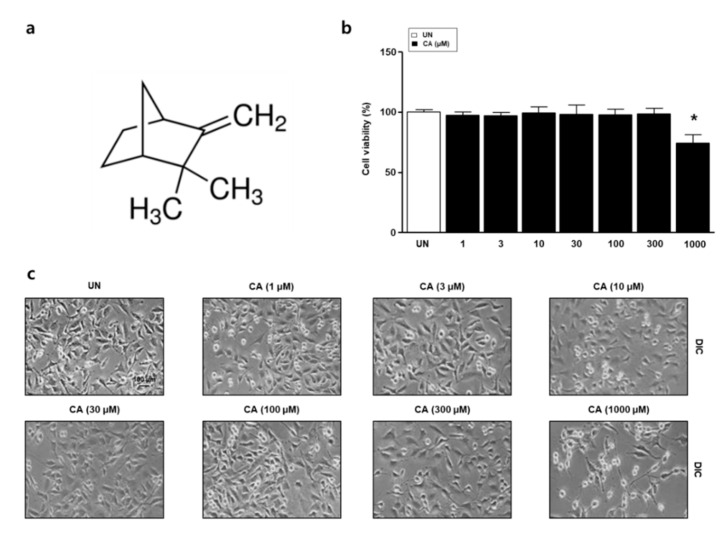
Effects of camphene (CA) on L6 skeletal muscle cells. (**a**) Chemical structure of CA; (**b**) L6 skeletal muscle cells were treated with CA (1, 3, 10, 30, 100, 300, and 1000 μM) and viability was measured by 2,3-bis-(2-methoxy-4-nitro-5-sulfophenyl)-2*H*-tetrazolium-5-carboxanilide (XTT) assay. The data are expressed as mean percentages relative to the untreated group ± standard deviations. * *p <* 0.05 vs. untreated group; (**c**) L6 cell morphology was examined by light microscopy.

To examine cell viability and morphology in response to camphene, we performed XTT assays and observed the cells by microscopy. L6 skeletal muscle cells were treated with camphene (1, 3, 10, 30, 100, 300, and 1000 μM), as in Figure 1a for 24 h. Viability and morphology were unaffected at concentrations ≤ 300 μM, as in Figure 1b,c. Therefore, 300 μM camphene was used in subsequent experiments.

### 3.2. Camphene Reduces the Starvation-Induced Oxidative Stress and Atrophy in L6 Skeletal Muscle Cells

**Figure 2:**
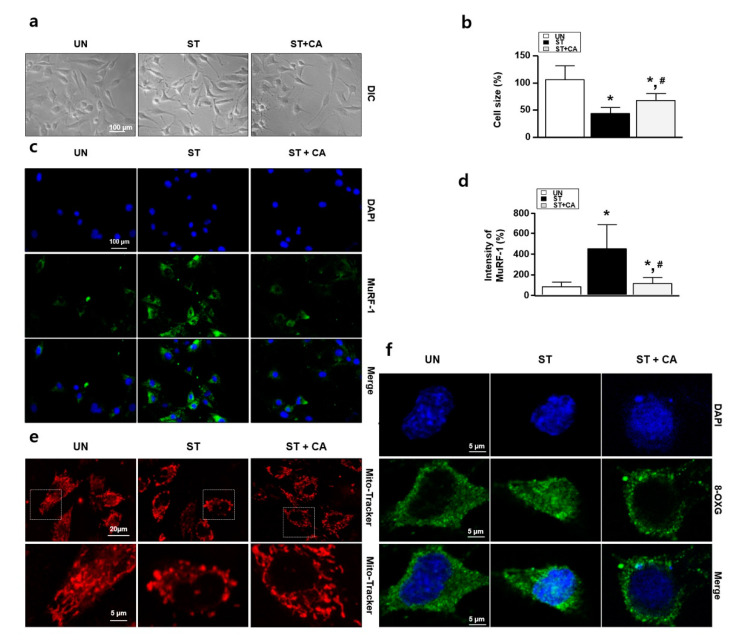
Effects of camphene on serum-starved L6 skeletal muscle cells. L6 skeletal muscle cells were cultured in Dulbecco’s modified Eagle’s medium (DMEM) supplemented with horse serum (1%) and treated with and without camphene (300 μM) for 3 d. UN: untreated group; ST: starvation group; ST + CA: starvation plus camphene group. (**a**) Observation of cell morphology by light microscopy; (**b**) the bar graph represents the relative cell size compared with the UN; (**c**) muscle RING-finger-1 (MuRF-1) expression (green color) was determined by immunocytochemistry; (**d**) the bar graph represents the relative intensity of MuRF-1; (**e**) immunocytochemistry assay with Mito Tracker^TM^ Red FM (red color); (**f**) immunocytochemistry assay with anti-8-oxoguanine (8-OXG) antibody (green color). DAPI, 4′-6-diamidino-2-phenylindole (blue color); data are (**b**,**d**) expressed as mean relative percentages compared with the UN ± standard deviations. * *p <* 0.05 vs. UN; ^#^ *p <* 0.05 vs. ST.

To investigate whether camphene can regulate serum starvation-induced skeletal muscle cell atrophy, we observed cell morphology by light and fluorescence microscopy and performed immunocytochemistry assays for MuRF-1. Figure 2a shows representative images of the morphological changes in L6 skeletal muscle cells after the treatment of starved cells (grown in DMEM supplemented with 1% horse serum) with 300 μM camphene for 3 d. The starved condition reduced the L6 cell size. In contrast, camphene treatment shows the reversal of the aberrant cell size and morphology in the starved condition, as in Figure 2b. Next, we performed the immunostaining using the anti-MuRF-1. The starvation increased the protein expression of MuRF-1, while the MuRF-1 expression decreased following camphene treatment, as in Figure 2c,d. We also performed immunocytochemistry assay with Mito Tracker^TM^ Red FM and anti-8-OXG antibody. Next, we tried to confirm whether ROS occurs in the atrophy of starvation-induced L6 cells. Excessive ROS generation in cells can alter the shape of mitochondria and increase 8-OXG expression. As shown in Figure 2e,f, mitochondrial morphology change and nuclear 8-OXG expression in L6 cells was increased upon starvation, whereas camphene treatment significantly reversed these effects of starvation-induced oxidative stress.

### 3.3. Camphene Reduces Hydrogen Peroxide (H_2_O_2_)-Induced Oxidative Stress in L6 Skeletal Muscle Cells 

**Figure 3:**
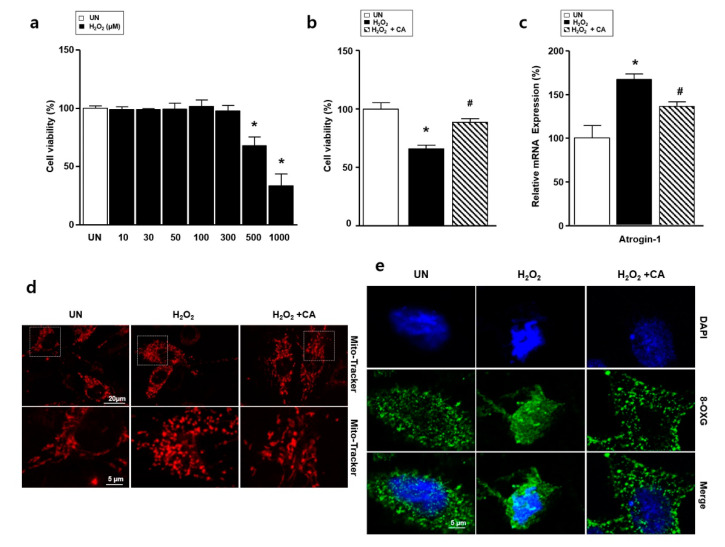
Effects of camphene (CA) on H_2_O_2_–induced atrophy of L6 skeletal muscle cells. L6 skeletal muscle cells were cultured in Dulbecco’s modified Eagle’s medium (DMEM) supplemented with horse serum (1%) and treated with H_2_O_2_ (500 μM) in the presence or absence of camphene (300 μM) for 24 h. UN: untreated group; H_2_O_2_: the group is treated with H_2_O_2_; H_2_O_2_ + CA: the group is treated with camphene and H_2_O_2_. (**a**) Effects of various H_2_O_2_ concentrations on L6 cell viability; (**b**) L6 cell viability in the UN, H_2_O_2_, and H_2_O_2_ + CA samples; (**c**) expression of atrogin-1 mRNA, expression levels were normalized to β-actin; (**d**) immunocytochemistry assay with Mito Tracker^TM^ Red FM (red color); (**e**) immunocytochemistry assay with 8-OXG antibody (green color). DAPI, 4′,6-diamidino-2-phenylindole; expression of lipid and carbohydrate metabolism genes in the UN, H_2_O_2_, and H_2_O_2_ + CA samples; all data are expressed as mean relative percentages compared with the UN ± standard deviations. * *p <* 0.05 vs. UN; ^#^ *p <* 0.05 vs. H_2_O_2_.

To measure the effects of H_2_O_2_-induced oxidative stress on L6 skeletal muscle cells, we treated them with H_2_O_2_ and performed XTT assays, immunocytochemistry assay, and real-time PCR. As the concentration of H_2_O_2_ increased, cell viability decreased dose-dependently, by 69.1 ± 5.3% (*p <* 0.01) and 33.4 ± 10.2% (*p <* 0.01) at 500 and 1000 μM, respectively, as in Figure 3a. Therefore, in subsequent experiments, we considered 500 μM H_2_O_2_ to be the half-maximal inhibitory concentration. L6 cells were pretreated with camphene (300 μM) for 1 h, then co-incubated with H_2_O_2_ for an additional 23 h to determine the effects of camphene on H_2_O_2_-mediated cell death. Treating L6 cells with camphene and H_2_O_2_ resulted in decreased cytotoxicity, as in Figure 3b. Next, we performed the mRNA expression analysis of atrogin-1 using real-time PCR. The H_2_O_2_ increased the expression of atrogin-1 compared with the UN, while the elevated atrogin-1 expression decreased following camphene treatment, as shown in Figure 3c. To investigate whether camphene could regulate H_2_O_2_-induced oxidative stress, Mito Tracker^TM^ Red FM and 8-OXG antibody staining were carried out. Mitochondrial morphology showed the spherical form after treatment with H_2_O_2_ in L6 cells. In contrast, treatment with camphene (300 μM) in the presence of H_2_O_2_ did not alter mitochondrial fission, as in Figure 3d. Furthermore, H_2_O_2_ treatment increased 8-OXG intensity in L6 cell nucleolus, which was significantly reduced by camphene, as in Figure 3e. 

### 3.4. Camphene Inhibits Starvation-Induced Skeletal Muscle Atrophy in Rats

**Figure 4:**
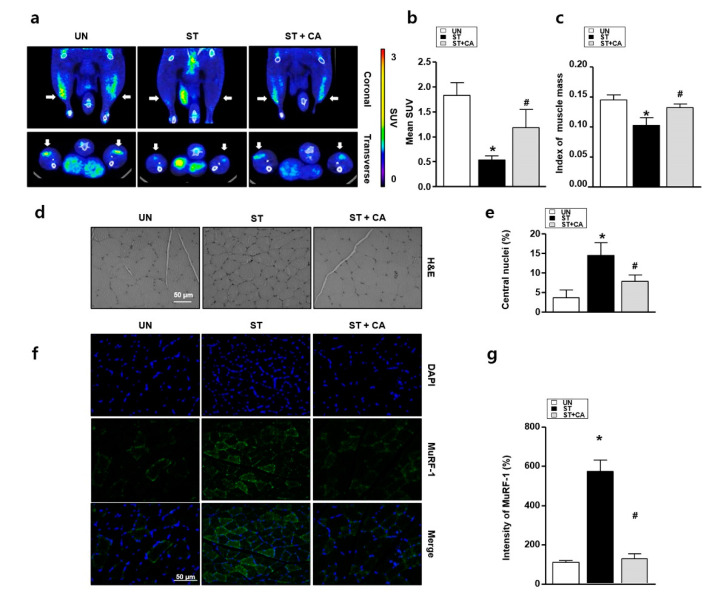
Effects of camphene on skeletal muscle cells in starved rats. UN: untreated group; ST: starved group; ST + CA: the group is starved and treated with camphene. (**a**) Representative ^18^F-fluoro-2-deoxy-D-glucose-positron emission tomography/computed tomography (^18^F-FDG-PET/CT) images of a rat model of skeletal muscle atrophy with and without camphene treatment; (**b**) mean standardized uptake value (SUV) values from ^18^F-FDG-PET examinations are shown (*n* = 4). * *p <* 0.05; (**c**) the index of muscle mass was defined as the ratio between the thickness of the muscle and length of the tibia acquired by MIM software; (**d**) hematoxylin and eosin staining of gastrocnemius muscles from each group; (**e**) the graph of the central nuclei ratio shown in panel d (*n* = 4); (**f**) representative MuRF-1 protein expression images in each group; (**g**) the bar graph indicates MuRF-1 protein expression (*n* = 4); * *p <* 0.05 vs. UN; ^#^ *p <* 0.05 vs. ST; all data are expressed as mean percentages relative to the UN ± standard deviations.

To investigate whether camphene can regulate starvation-induced muscle atrophy in vivo, we performed ^18^F-FDG PET/CT, Haemotoxylin and Eosin (H&E) staining and immunohistochemistry assay. With prolonged starvation, skeletal muscle cells display increased ROS-induced oxidative stress, and muscle size decreases [18]. Thus, we examined the protective effects of camphene against starvation-induced skeletal muscle atrophy. By ^18^F-FDG PET/CT, the mean SUVs of the UN, ST, and ST + CA were significantly different, at 1.78 ± 0.29, 0.56 ± 0.11, and 1.12 ± 0.46, respectively (*p* = 0.016). Post hoc analysis showed that the UN and ST + CA had significantly higher SUVs than the ST (*p* < 0.05). However, there was no difference in SUV between the UN and ST + CA (*p* > 0.05; Figure 4a,b). Next, to determine the protective effect of camphene starvation-induced skeletal muscle atrophy in rats, muscle mass measurements using CT with the index of muscle mass (IMM) were performed. IMM in the ST was significantly reduced compared with that of the UN, while the ST + CA was similar to that of the UN, as in Figure 4c. Hematoxylin and eosin staining was also performed to examine the histological change of muscle morphology. The stained specimens were obtained from muscle sections and then analyzed by counting the central nuclei of the muscles in the magnification fields. As shown in Figure 4d,e, the number of central nuclei in ST is greater than that in UN. In the ST + CA, the skeletal muscle cells’ central nuclei were significantly reduced compared with that of the ST. The presence of MuRF-1 indicates the prior degeneration and persistent atrophy of the muscle [24]. To confirm muscle atrophy, we performed an immunohistochemistry assay with MuRF-1 antibody. The expression of MuRF-1 in the ST significantly increased compared with that in the UN, while MuRF-1 expression in the ST + CA was similar in the UN and ST + CA, as in Figure 4f,g.

### 3.5. The Effect of Camphene on Lipid Metabolism In Vitro and In Vivo

**Figure 5:**
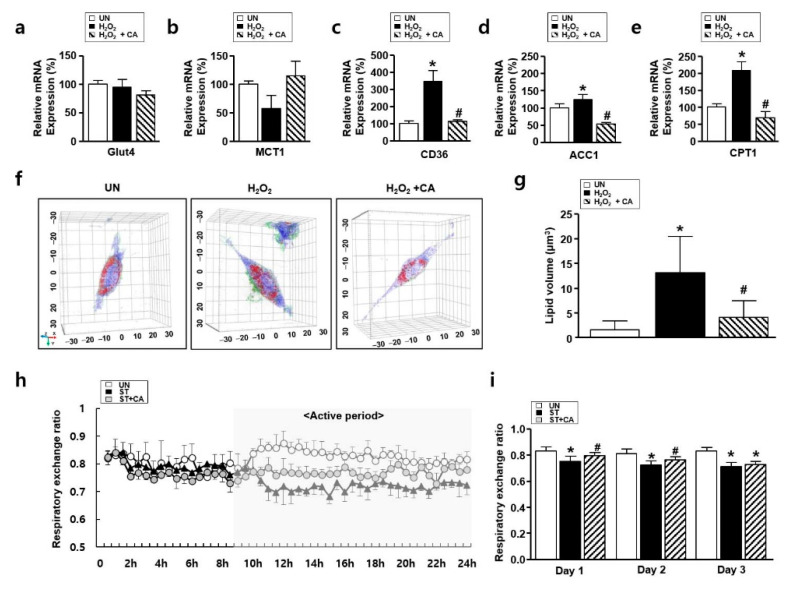
The effect of camphene on lipid metabolism in hydrogen peroxide (H_2_O_2_)-stimulated L6 skeletal muscle cells and starved rats. (**a**–**e**) Expression of carbohydrate and lipid metabolism genes in the UN, H_2_O_2_, and H_2_O_2_ + CA. Expression levels were normalized to β-actin. * *p <* 0.05 vs. UN; ^#^ *p <* 0.05 vs. H_2_O_2_; (**f**,**g**) tomographic images of L6 cells treated with H_2_O_2_ with and without camphene. Red indicates lipids; UN: untreated group; H_2_O_2_: the group is treated with H_2_O_2_ (500 μM) for 24 h; H_2_O_2_ + CA: the group is treated with camphene (300 μM) and H_2_O_2_ (500 μM) for 24 h. * *p <* 0.05 vs. UN; ^#^ *p <* 0.05 vs. H_2_O_2_; (**h**,**i**) respiratory exchange ratios of the UN, ST, and ST + CA over (**h**) 24 h and (**i**) change in mean respiratory exchange rate over 3 days (*n* = 8). UN: untreated group; ST: starved group; ST + CA: the group is starved and treated with camphene. * *p <* 0.05 vs. UN; ^#^ *p <* 0.05 vs. ST; all data are expressed as mean percentages relative to the UN ± standard deviations.

ROS alters in glucose and lipid metabolism [25]. Especially, elevated lipid metabolism is involved in the progression of atrophy [11]. To explore lipid metabolism in vitro and in vivo atrophy models, we preformed holotomographic microscopy, real-time PCR, and respiratory gas analysis, respectively. First, to explore the relationships between carbohydrate/lipid metabolism and atrophy, an analysis of mRNA expression was performed. Indices of glucose metabolism, such as Glut4 and MCT1, were not altered for each group. H_2_O_2_ treatment resulted in overexpression of lipid metabolism indicators, such as ACC1, CD36, and CPT1. However, these indicators decreased dramatically after treatment with camphene, as in Figure 5a–e. Next, to confirm the effect of camphene on H_2_O_2_-induced lipid metabolism, holotomographic observation was performed. Cell morphology was observed under a holotomographic microscopy, as in a previous study [26]. Holotomographic microscopy revealed lipid accumulation after H_2_O_2_ treatment, which was also decreased by camphene, as in Figure 5f,g. Next, a respiratory gas analysis was performed to determine whether camphene contributed to the regulation of lipid metabolism during atrophy. Figure 5h shows the respiratory exchange ratios (RERs) in each group during the first day of fasting. The RER followed the circadian rhythm in all groups. Interestingly, there were no differences between any groups during the non-active (light) period, when the rats were asleep. However, with the onset of activity (in the dark period), the UN mainly oxidized carbohydrates, while the two starvation groups (ST and ST + CA) primarily oxidized fat. Figure 5i shows the mean RERs in the same metabolic chamber over 3 d. On the first day, the RER was lower in both ST compared to the UN (*p <* 0.05). Besides, the ST + CA had a significantly higher RER than the ST. A similar difference was observed on the second day, with a significantly higher RER in the ST + CA than in the ST. This trend continued on the third day; however, the difference was not statistically significant. 

## 4. Discussion

We have demonstrated that camphene regulates skeletal muscle atrophy in vitro and in vivo. Excessive ROS levels due to exogenous and endogenous factors are etiologically involved in skeletal muscle atrophy [27]. ROS induces muscle-specific E3 ubiquitin ligases, such as MuRF-1 and atrogin-1, eventually leading to decreased muscle energy efficiency and increased muscle atrophy [28]. The properties of camphene have been reported to have excellent antioxidant activity both in vitro and in vivo [29]. We also found that camphene, a monoterpene, had an inhibitory effect on starvation-induced ROS generation and skeletal muscle atrophy, as in Figure 2 and Figure 3. Furthermore, we confirmed that the inhibitory effects of camphene on skeletal muscle atrophy, in Figure 4, occurred through lipid metabolism regulation, as in Figure 5. Muscle atrophy is caused by muscle loss and elevated lipid accumulation [30]. Our findings demonstrate that camphene treatment regulates lipid metabolism in skeletal muscle and decreases the expression of MuRF-1, atrogin-1, and lipid metabolic enzymes, such as ACC1, CD36, and CPT1 during ROS-induced muscle atrophy. To the best of our knowledge, central-nucleated myofibers and regenerative fibers are increased in atrophic mouse models. In this study, we found that the number of increased central nuclei in the atrophy model was decreased by camphene treatment. These results imply that camphene regulates ROS generation, muscle atrophy, and energy metabolism under nutrient-starved conditions. Therefore, we suggest that camphene have protective effects against the ROS-induced muscle atrophy. 

Etiologically, progressive and severe diseases are primarily caused by increased abnormal ROS production, decreased energy productivity, and impaired self-defense function [31]. In particular, muscle atrophy is associated with increased ROS production due to a variety of pathogenic conditions leading to chronic metabolic disorders, such as inflammation, diabetes, and cancer [32]. Therefore, the cause of muscular atrophy is defined as the main cause of unremoved ROS, which induces oxidative stress and changes homeostasis in vivo. Diverse studies have reported that mitochondrial dysfunction and ROS generation are key mechanisms in the progression of muscle atrophy [33]. These results are based on the fact that ROS-related oxidative stress is a mediator of atrophy from its onset. We found that muscle atrophy is similar under nutrient-starvation and in the presence of H_2_O_2_, as in Figure 3. We speculated that the oxidative stress-induced muscle atrophy was triggered by mitochondrial dysfunction and altered morphology, and increased nuclear expression of 8-OXG. As camphene treatment did not alter the extent of mitochondria shape or translocalized 8-OXG into the nucleus, camphene regulates oxidative stress caused by ROS and, consequently, muscle atrophy, as in Figure 2 and Figure 3. Ábrigo et al. [28] have been reported that regulation of ROS can affect muscle atrophy. In the present study, we found that camphene regulates oxidative stress and muscle dysfunction caused by ROS. Therefore, we suggest that camphene may help mitigate muscle atrophy due to sarcopenia and cachexia.

Sarcopenia inevitably occurs with aging and reportedly increases in those with sedentary lifestyles and decreased physical activity [34]. A recent study reported that lipid accumulation occurs when atrophy begins. Fukawa et al. [35] reported that ingestion and regulation of unsaturated fatty acids could affect muscle atrophy. Notably, the hypothesis that skeletal muscle lipid regulation could be a major mechanism for suppressing atrophy has attracted attention [36]. Our holotomographic microscopy results were consistent with the observed changes in the mRNA levels of genes involved in lipid metabolism, as in Figure 5a–g. Besides, we found that the camphene treatment of starved rats resulted in a higher RER than that observed in untreated starved rats, as in Figure 5h,i. The RER can be expressed as the ratio of O_2_ uptake to CO_2_ emission. The closer it is to 0.7, the more lipids are used as the main energy source, and the closer it is to 1, the more carbohydrates are used as the main energy source [37]. This study found that starvation promoted fat oxidation in all starved groups compared to the untreated group. However, during starvation, camphene treatment inhibited fat oxidation, as in Figure 5h,i. This finding was consistent with in vitro studies, in Figure 5a–g, and indicates that camphene treatment prevents ROS increases. Therefore, our results also support the hypothesis by Vallianou et al. [17], suggesting that camphene inhibits atrophy by regulating lipid metabolism.

In addition, our investigations have been expanded to the diagnosis of muscle attributes, such as muscle mass and physical performance in starvation-induced muscle atrophy. In this study, we confirmed muscle function by imaging glucose metabolism using ^18^F-FDG PET/CT and measured muscle mass through CT, as in Figure 4c. Muscle uptake of ^18^F-FDG was significantly reduced in starved animals, and this effect was rescued by camphene treatment. The results showed that the progression of atrophy and changes in glucose/lipid metabolism in muscles are closely related, as in Figure 3 and Figure 4. Therefore, we suggest that ^18^F-FDG PET/CT imaging in a fasted state may be an important tool for diagnosing sarcopenia and evaluating treatment effects.

## 5. Conclusions

The present study demonstrates that camphene attenuates ROS-related muscle atrophy. Camphene significantly inhibits ROS-related oxidative stress and MuRF-1 and atrogin-1 expression, and also regulates lipid metabolism indicators, such as ACC1, CD36, and CPT1. In vivo, camphene diminished muscle dysfunctions, such as reduced muscle mass and glucose uptake. Therefore, camphene may be a promising therapeutic agent in the treatment of sarcopenia and cachexia. However, our experiment demonstrated the effectiveness of camphene by conducting experiments on muscle atrophy using the starvation model for 48 h. Of note, sarcopenia is a chronic atrophy program that is fundamentally different from acute starvation. Therefore, further studies using an animal model in which muscle atrophy occurs due to aging is necessary.

## Figures and Tables

**Table 1:** Primer sequences for real-time PCR.

Gene Product	Primer Sequence
Glut4	Forward: 5′-AGAGTCTAAAGCGCCT-3′
	Reverse: 5′- CCGAGACCAACGTGAA-3′
ACC1	Forward: 5′-AGGAAGATGGTGTCCCGCTCTG-3′
	Reverse: 5′-GGGGAGATGTGCTGGGTCAT-3′
MCT1	Forward: 5′-AGAAGTCAGCCTTCCTCCTTT-3′
	Reverse: 5′-CCACAAGCCCAGTATGTGTAT-3′
CD36	Forward: 5′-CGGCGATGAGAAAGCAGA-3′
	Reverse: 5′-ACTCCAACACCAAGTAAGACCA-3′
CPT1	Forward: 5′-GTGCTGGAGGTGGCTTTGGT-3′
	Reverse: 5′-TGCTTGACGGATGTGGTTCC-3′
Atrogin-1	Forward: 5′-GAACATCATGCAGAGGCTGA-3′
	Reverse: 5′-GTAGCCGGTCTTCACTGAGC-3′
β-actin	Forward: 5′-GGCCAACCGTGAAAAGATG-3′
	Reverse: 5′-GGATCTTCATGAGGTAGTCTGTC-3′
